# Can Chinese patients restore normal knee function 1 year after total knee arthroplasty?

**DOI:** 10.3389/fmed.2025.1683601

**Published:** 2025-11-17

**Authors:** YiShun Guo, HaoDong Wu, XiaoFeng Chang, HuanLi Bao, JianBing Ma, Chao Xu, ShuXin Yao

**Affiliations:** 1 Department of Knee Joint Surgery, Honghui Hospital, Xi’an Jiaotong University, Xi’an, China; 2 Graduate Student Work Department, Xi’an Medical University, Xi’an, China; 3 Medical College of Yan’an University, Yan’an University, Yan’an, China

**Keywords:** total knee arthroplasty, knee osteoarthritis, functional recovery, knee function assessment, localized evaluation protocols, cross-sectional study

## Abstract

**Objective:**

To develop a culturally adapted assessment tool for evaluating one-year postoperative knee function in Chinese patients after total knee arthroplasty (TKA), and to establish foundations for localized evaluation protocols and early postoperative interventions.

**Methods:**

This cross-sectional study systematically reviewed knee function assessment items for post-TKA patients. Based on expert and patient focus group discussions, a set of evaluation items was established, followed by designing a self-administered questionnaire assessing importance, frequency, and difficulty. From February to December 2024, TKA patients at 1 year post-surgery and age-matched healthy controls were recruited at Honghui Hospital, Xi’an, China. High-frequency activities were identified and compared between groups.

**Results:**

A total of 39 culturally relevant knee function items were developed through expert and patient consensus. The study included 713 TKA patients and 675 age-matched healthy controls, and 14 high-frequency activities were identified based on importance ratings. No significant functional differences were observed between TKA patients and controls in individuals under 65 years. In the 65–75-year group, only females showed decreased performance in picking up items from the ground (*p* < 0.001). Among participants older than 75 years, males showed significant deficits in putting on and taking off shoes and socks (*p* = 0.008), squatting (*p* < 0.001), picking up items from the ground (*p* = 0.009), and carrying heavy objects with one hand (*p* < 0.001), while females demonstrated reduced ability in bathing and wiping (*p* < 0.001), picking up items from the ground (*p* < 0.001), and squatting (*p* = 0.048).

**Conclusion:**

Most patients regain daily function 1 year after TKA, but limitations persisted in older patients. The 14 culturally relevant activities identified provide a practical basis for a localized assessment. Future work will involve longitudinal validation and clinical integration to support individualized postoperative rehabilitation.

## Introduction

Total knee arthroplasty (TKA) is an effective treatment for end-stage knee osteoarthritis (KOA), significantly reducing pain while improving function and quality of life (1). Nevertheless, approximately 20% of patients experience dissatisfaction with postoperative functional outcomes (2), suggesting that traditional evaluation systems—predominantly reliant on objective metrics and clinician assessments—may fail to identify key determinants of patient satisfaction. Therefore, aligning patients’ perceived functional recovery with their preoperative expectations is essential for optimizing post-TKA satisfaction (3).

The contemporary medical model prioritizes patient-centered care, shifting clinical evaluation from clinician-centered biomedical metrics to multidimensional assessments incorporating psychological, social, and quality-of-life domains (4). Validated patient-reported outcome measures (PROMs), which include symptoms, mental health, social functioning, and overall well-being, are therefore essential. Systematic evaluation of postoperative functional recovery provides critical insight for optimizing outcomes, improving patient experience, and guiding rehabilitation strategies (5).

Three major limitations persist in current post-TKA functional assessment tools: Commonly used instruments such as the new-KSS (6) and WOMAC (7) were developed in Western populations. Due to cultural and lifestyle differences, these tools exhibit low completion rates among Chinese patients and fail to capture functionally relevant activities, particularly those of specific concern to this population (8). Neglect of Age-Related Decline: Existing tools fail to adjust for physiological musculoskeletal deterioration in elderly patients (9). Moreover, according to the COSMIN criteria, several widely used scales contain structural flaws that compromise measurement objectivity and scientific rigor (10). Lack of Goal Individualization: While functional recovery expectations are modulated by personal factors (activity demands, occupation, exercise habits, and self-perception), current evaluations overemphasize basic tasks without incorporating patient-specific goals or age-adjusted benchmarks. This misalignment contributes to discordant clinician-patient expectations (11). Surgeons lack standardized protocols for postoperative goal-setting, whereas patients frequently pursue unrealistic pre-morbid functional states—overlooking disease chronicity, aging, and comorbidities (12).

Current functional evaluation systems lack cultural and population specificity for Chinese TKA patients, limiting their accuracy in reflecting true postoperative function. This hinders personalized rehabilitation planning, optimization of interventions, and ultimately patient satisfaction (13).

This study aimed to develop a culturally adapted item pool for evaluating knee function in Chinese patients. By systematically identifying daily functional tasks relevant to Chinese individuals, we established assessment items that reflect high-frequency and culturally specific activities. We hypothesized that 1 year after TKA, most patients would recover to a functional level comparable to healthy individuals, although differences may exist across age and sex subgroups. This study provides insight into the one-year postoperative recovery status of TKA patients, offering a scientific basis for functional outcome evaluation and supporting individualized rehabilitation planning.

## Materials and methods

### Development and refinement of the item pool

A comprehensive literature search was conducted across the following databases: PubMed, Embase, Web of Science, Scopus, China National Knowledge Infrastructure (CNKI), Wanfang Data Knowledge Service Platform (Wan Fang), and VIP Chinese Journal Service Platform (VIP). To systematically identify psychometrically validated patient-reported outcome measures (PROMs) for individuals undergoing TKA, the search strategy included combinations of keywords across three domains: (1) patient-reported outcome measures; (2) measurement properties (reliability, validity, internal consistency, responsiveness, measurement error, and minimal clinically important difference); and (3) TKA. The PubMed search strategy followed Wang et al. (4). Studies were included if they were full-text articles in English or Chinese reporting development or evaluation of PROMs specific to TKA populations. Exclusion criteria were: (1) lack of measurement property evaluation; (2) non-TKA populations; and (3) incomplete reports.

After removing duplicates, three reviewers independently screened and verified eligible studies. Knee function assessment items meeting inclusion criteria were extracted to develop an initial item pool, supplemented with relevant items from the CHARLS database, which contains comprehensive data on the Chinese elderly population (14).

The item pool underwent a 60-min one-on-one interview with a TKA expert, who reviewed the items and provided feedback on wording, organization, and content, while discussing the impact of TKA on daily life and other questionnaire aspects (length, response options). Purposeful sampling was used to recruit TKA patients for a 90-min face-to-face focus group and subsequent individual interviews conducted on January 15, 2024, at Honghui Hospital. Participants discussed the relevance and structure of the item pool and described how their condition affects daily activities.

### Questionnaire design and data collection

This study employed a cross-sectional design. After establishing a preliminary item pool for knee function assessment, we developed a self-administered questionnaire to evaluate three dimensions (15): the perceived importance of knee-related activities, the frequency of engagement in these activities, and the difficulty of performing these activities due to knee problems.

Between February and December 2024, trained research personnel recruited participants from Xi’an Honghui Hospital, including patients 1 year after TKA and age-matched healthy controls. Participants completed the questionnaire via the Wenjuanxing platform or paper-based. All research personnel received standardized training in communication, coordination, interview procedures, and data quality control, and verified each questionnaire for completeness and accuracy at the time of submission to ensure data integrity. All patients had previously undergone TKA surgery at Xi’an Honghui Hospital. This study involving human participants was reviewed and approved by the Ethics Committee of Xi’an Jiaotong University Affiliated Honghui Hospital on January 15, 2024 (202401012). All participants provided written informed consent prior to participation. The study was conducted in accordance with the principles of the Declaration of Helsinki.

The questionnaire comprised two sections: (1) general demographic information, including age, sex, height, weight, educational level, long-term living conditions, and residence; and (2) a series of activity-specific items derived from the knee function item pool. Each activity was evaluated across three dimensions: its perceived importance in daily life, frequency of performance, and the level of knee-related difficulty in completing the task. Participants were instructed to rate each activity based on their real-world experiences.

Both TKA patients and healthy controls were required to assess and rate each activity in terms of: Importance (1 = Not important, 2 = Slightly important, 3 = Moderately important, 4 = Very important, 5 = Extremely important), Frequency (1 = Never, 2 = Rarely, 3 = Occasionally, 4 = Often, 5 = Daily), Difficulty (1 = No difficulty, 2 = Slight difficulty, 3 = Moderate difficulty, 4 = Severe difficulty, 5 = Extreme difficulty).

### Criteria for selecting high-frequency activities and functional grouping assessment

Selection Criteria for High-Frequency Activities: Each item in the patient questionnaire was assigned an importance score, expressed as Mean ± SD. Items were classified as high-frequency activities if they met both of the following criteria: (1) mean importance score >3.5; and (2) coefficient of variation (CV) < 0.25. These thresholds are widely used in Delphi and instrument-development studies to ensure item relevance and consensus (16). Functional Recovery Assessment: Differences in difficulty scores between the TKA group and healthy controls were analyzed using the independent-samples Mann–Whitney *U* test. Subgroup analyses were performed stratified by age (<65 years, 65–75 years, and >75 years) (17) and sex. Scores, treated as ordinal categorical variables ranging from 1 to 5, were compared. *p*-value < 0.05 was considered statistically significant.

### Statistical analyses

All statistical analyses were performed using SPSS version 26.0. Continuous variables were presented as Mean ± SD, and categorical variables were reported as frequencies and percentages (*n*, %). Non-parametric tests were used for non-normally distributed data, while independent-samples t-tests were applied to normally distributed variables. Sample size was estimated *a priori* using G*Power (effect size *d* = 0.5, *α* = 0.05, power = 0.80), requiring at least 128 participants per group.

## Results

### Literature search and initial item development

**Figure 1:**
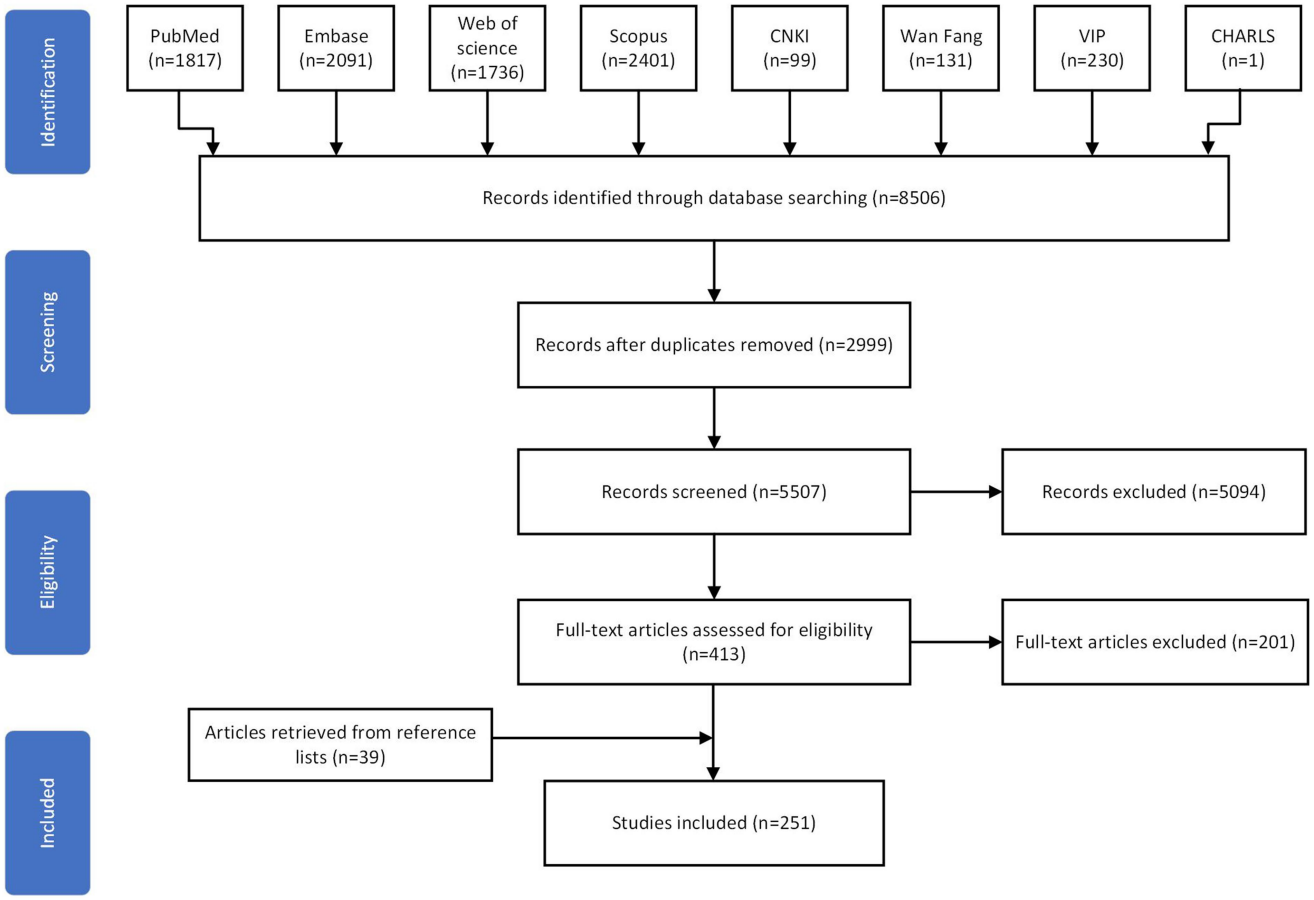
Flowchart of the study selection process.

A total of 8,506 articles were initially identified through systematic keyword searches, of which 251 met the inclusion criteria (Figure 1). Based on the extracted functional items from these studies, an initial pool of 69 knee function assessment items was compiled (5–7, 14, 18–25).

### Cross-cultural adaptation and item selection

Most identified items originated from Western-developed scales based on Western cultural norms and lifestyles. The applicability of cross-culturally adapted Chinese versions to Chinese populations remains controversial (26). For instance, items like “getting in and out of a bathtub” from the WOMAC scale and high-level recreational activities (golf, bowling, gardening) in the KSS scale may not align with Chinese living conditions and culture, potentially affecting measurement validity (27). Sociocultural factors also influence patients’ functional priorities. Chinese TKA patients generally place greater emphasis on self-care and basic functional independence, while attaching less importance to social or sports-related activities (28).

A panel of 12 joint surgery and rehabilitation experts conducted the first-round screening of 69 items. Through 60-min interviews with TKA specialists, the panel reviewed wording, organization, and questionnaire structure. Items that were inconsistent with typical Chinese lifestyles, such as “playing golf,” “getting in and out of a bathtub,” and “skiing,” were removed, resulting in 41 retained items consisting of 18 basic and 23 advanced activities.

To further ensure that the selected items were representative of the functional demands of Chinese TKA patients, patient focus groups were conducted. Recruited patients participated in face-to-face group discussions and follow-up interviews for a second round of item refinement. During this process, low-frequency advanced activities such as “impact sports” and “cutting toenails” were removed (19, 29).

### Self-administered questionnaire design

After two rounds of screening, a total of 39 assessment items were finalized, including 18 basic functional activities and 21 advanced functional activities (Table 1). Based on these selected items, a three-dimensional self-administered questionnaire was developed. This questionnaire evaluates, for each activity, the importance of the activity, the frequency of participation, and the difficulty experienced in performing the activity due to knee involvement.

**Table 1:** Sources and screening results of the item pool.

Scales and literature		Evaluation items
ICF Core Set Activity and Participation Dimension for Osteoarthritis (knee-related items) [20]; New-KSS [11]; WOMAC [12]; KOOS [14]; OKS [13]; HSS [15]; The most concerned movement functions before and after surgery for TKA patients [6, 21–23]; China Health and Retirement Longitudinal Study (CHARLS) (69 items)		Overall ability to get around, Taking off socks or stockings, Weight bearing, Walking on a flat surface, Walking on rough terrain, Stairs, Squatting, Running, Jogging, Jumping, Sitting, Light Household activity (sweep, vacuum, cleaning), Heavy domestic duties (Shoveling snow, scrubbing floor), Stepping over objects, Riding a bicycle, Bath/shower, Standing up from a low sitting position, Using public transportation, Standing. Gardening, Get in (out) of a car, Sitting on/rising from the floor, Tennis, Skiing, Farming, Hill walking, Walking up and down an incline, Kneeling, Cross-legged sitting, Bending to floor, Entering and exiting bathtubs, Going shopping, Rising from bed, Getting on (off) toilet, stepping to the side, Turning or twisting on the knee. Swimming, Golfing, Bowling. Distance walking, Dancing, Stretching, Weight-lifting, Leg-extensions, Stationary biking, Walking with aids (Reduced items), Leg press, Elliptical trainer, Aerobic exercises, Making sharp turns while running fast, Rolling over in bed, Taking care of children, Moving heavy objects, Cutting toe-nails, Kneel down and get up again afterward, Gym workout, Walking on slippery surface, Impact sports, Operating foot pedals, Carrying, Pushing, Pulling, Clambering, Using a squat toilet, Single-leg standing, Dancing in the square, playing table tennis, traditional activities (practicing Tai Chi, doing fitness exercises, sword dancing).
The selected items after discussion by the research team (41 evaluation items)	Basic items (18 items)	Continuous walking on flat ground, Walking up and down an incline, Walking on rough terrain, Carrying heavy objects with one hand, Going up and down stairs, Squatting, Sitting toilet use, Using a squat toilet, Standing, Sitting, cross-legged sitting, Putting on and taking off shoes and socks, Picking up items from the ground, kneeling, Unaided rise from a low position without handrail, Bathing and wiping, holding a baby or heavy objects with one hand, simple household chores
	Advanced items (23 items)	Climbing ladders, zigzagging back-and-forth movements, lateral stepping, light farm or gardening work, getting on and off busses, entering and exiting cars, driving a vehicle oneself, dancing in the square, swimming, traditional activities (Tai Chi, aerobics, sword dancing), stretching exercises (stretching lower limb muscles), muscle strength training (weight-bearing flexion and extension of joints), jogging, cycling, table tennis, badminton, basketball, standing on one leg, climbing mountains, single-foot or double-foot jumping, rotating or turning on one leg as the axis. Impact sports Cutting toenails
The selected items after discussion by the patient group (39 evaluation items)	Basic items (18 items)	Continuous walking on flat ground, Walking up and down an incline, Walking on rough terrain, Carrying heavy objects with one hand, Going up and down stairs, Squatting, Sitting toilet use, Using a squat toilet, Standing, Sitting, cross-legged sitting, Putting on and taking off shoes and socks, Picking up items from the ground, kneeling, Unaided rise from a low position without handrail, Bathing and wiping, holding a baby or heavy objects with one hand, simple household chores
	Advanced items (21 items)	Climbing ladders, zigzagging back-and-forth movements, lateral stepping, light farm or gardening work, getting on and off busses, entering and exiting cars, driving a vehicle oneself, dancing in the square, swimming, traditional activities (Tai Chi, aerobics, sword dancing), stretching exercises (stretching lower limb muscles), muscle strength training (weight-bearing flexion and extension of joints), jogging, cycling, table tennis, badminton, basketball, Single-leg standing, climbing mountains, single-foot or double-foot jumping, rotating or turning on one leg as the axis.

### Sample collection and demographic characteristics

Questionnaire data were collected from February to December 2024 at Xi’an Honghui Hospital. A total of 713 patients who were 1 year post–TKA and 675 healthy controls were enrolled (Table 2).

**Table 2:** Demographic characteristics.

Variables	Patients 1 year after TKA (*n* = 713)	Healthy controls (*n* = 675)
Age, years, (mean ± SD)	66.85 ± 7.04	65.94 ± 12.79
Female, *n* (%)	478 (67.04%)	448 (66.4%)
BMI, kg/m^2^	25.39 ± 3.51	25.05 ± 3.26
Education level, *n* (%)		
Primary school or less	224 (31.4%)	181 (26.8%)
Junior high school	213 (29.8%)	186 (27.5%)
High school	151 (21.2%)	167 (24.7%)
University or higher	125 (17.5%)	141 (20.8%)
Living arrangement, *n* (%)		
Living alone	47 (6.59%)	99 (14.6%)
Living with spouse	487 (68.3%)	447 (66.2%)
Living with child	179 (25.1%)	129 (19.1%)
Long-term residence, *n* (%)		
Urban	294 (41.2%)	221 (32.7%)
Rural	419 (58.7%)	454 (67.2%)

*BMI = body mass index; SD = standard deviation.

The TKA group had a mean age of 66 years, and females accounted for 67.04%, a significantly higher proportion than males. The mean BMI was 25.39 kg/m^2^. Among TKA patients, 61.2% had an education level of junior high school or below. In addition, 68.3% lived with a spouse, and 58.7% resided in urban areas.

The control group had a mean age of 65 years, with 55.9% females. The mean BMI was 25.05 kg/m^2^. Among them, 54.3% had education levels of junior high school or below, 66.2% lived long-term with a spouse, and 67.2% resided in urban areas.

### Identification of high-frequency functional activities based on importance scores

This study used both electronic and paper-based questionnaires to assess TKA patients’ functional performance across multiple activities. Each item was rated across three dimensions: perceived importance, frequency of performance, and difficulty due to knee impairment, using a 5-point Likert scale.

Data were processed and analyzed using SPSS version 26.0. For each item, the importance score was summarized as Mean ± SD, along with the percentage of participants assigning a full score (5 points). Based on the predefined selection criteria—mean importance score >3.5 and CV < 0.25—a total of 14 high-frequency functional activities were identified (Table 3).

**Table 3:** Importance scores, maximum score proportion, and CV for knee function evaluation items in TKA patients.

Evaluation items	Importance score	Maximum score proportion	CV	Evaluation items	Importance score	Maximum score proportion	CV
Standing	4.92 ± 0.38	95.4	0.077	Holding a baby or heavy objects with one hand	3.08 ± 1.48	45.9	0.479
Continuous walking on flat ground	4.81 ± 0.62	92.1	0.129	Zigzagging back-and-forth movements	3.05 ± 1.44	0.0	0.473
Putting on and taking off shoes and socks	4.73 ± 0.76	68.7	0.161	Cross-legged sitting	2.91 ± 1.45	12.7	0.499
Bathing and wiping	4.49 ± 0.89	65.7	0.198	Stretching exercises (stretching lower limb muscles)	2.84 ± 1.52	12.3	0.536
Sitting toilet use	4.36 ± 0.71	62.1	0.163	Muscle strength training (weight-bearing flexion and extension of joints)	2.77 ± 1.49	12.3	0.540
Simple household chores	4.29 ± 0.61	71.4	0.142	Jogging	2.63 ± 1.57	45.7	0.576
Unaided rise from a low position without handrail	4.17 ± 1.03	78.2	0.247	Single-leg standing	2.62 ± 1.53	56.8	0.583
Squatting	4.12 ± 1.01	51.3	0.245	Climbing ladders	2.41 ± 1.51	13.7	0.628
Picking up items from the ground	4.05 ± 0.97	49.3	0.240	Cycling	2.39 ± 1.53	16.3	0.643
Sitting	3.92 ± 0.57	53.4	0.145	Rotating or turning on one leg as the axis	2.26 ± 1.48	7.2	0.655
Walking on rough terrain	3.91 ± 0.87	67.5	0.223	Climbing mountains	2.21 ± 1.52	41.5	0.682
Walking up and down an incline	3.87 ± 0.93	54.3	0.240	Dancing in the square	2.17 ± 1.43	12.7	0.658
Going up and down stairs	3.65 ± 0.83	55.4	0.228	Tai Chi, aerobics, sword dancing	2.11 ± 1.46	56.7	0.692
Carrying heavy objects with one hand	3.53 ± 0.57	47.5	0.169	Single-foot or double-foot jumping	2.04 ± 1.53	18.3	0.749
Using a squat toilet	3.51 ± 1.41	39.2	0.416	Driving a vehicle oneself	2.04 ± 1.42	31.5	0.695
Entering and exiting cars	3.47 ± 1.22	58.2	0.363	Table tennis	1.83 ± 1.34	21.7	0.734
Getting on and off busses	3.31 ± 1.38	67.8	0.416	Swimming	1.72 ± 1.35	12.7	0.783
Lateral stepping	3.21 ± 1.40	17.1	0.435	Badminton	1.61 ± 1.26	0.0	0.784
Light farm or gardening work	3.09 ± 1.49	27.6	0.482	Basketball	1.54 ± 1.20	0.0	0.779
Kneeling	3.08 ± 1.52	21.8	0.494				

All 14 selected items were categorized as basic functional activities, including: Standing, Continuous walking on flat ground, Putting on and taking off shoes and socks, Bathing and wiping, Sitting toilet use, Simple household chores, Unaided rise from a low position without handrail, Squatting, Picking up items from the ground Sitting, Walking on rough terrain, Walking up and down an incline, Going up and down stairs, Carrying heavy objects with one hand.

### Subgroup analysis of high-frequency functional activities

After 14 high-frequency functional activities identified as most important for Chinese TKA patients, a comprehensive analysis was performed to examine the relationships among importance, frequency, and difficulty ratings across all participants.

**Figure 2:**
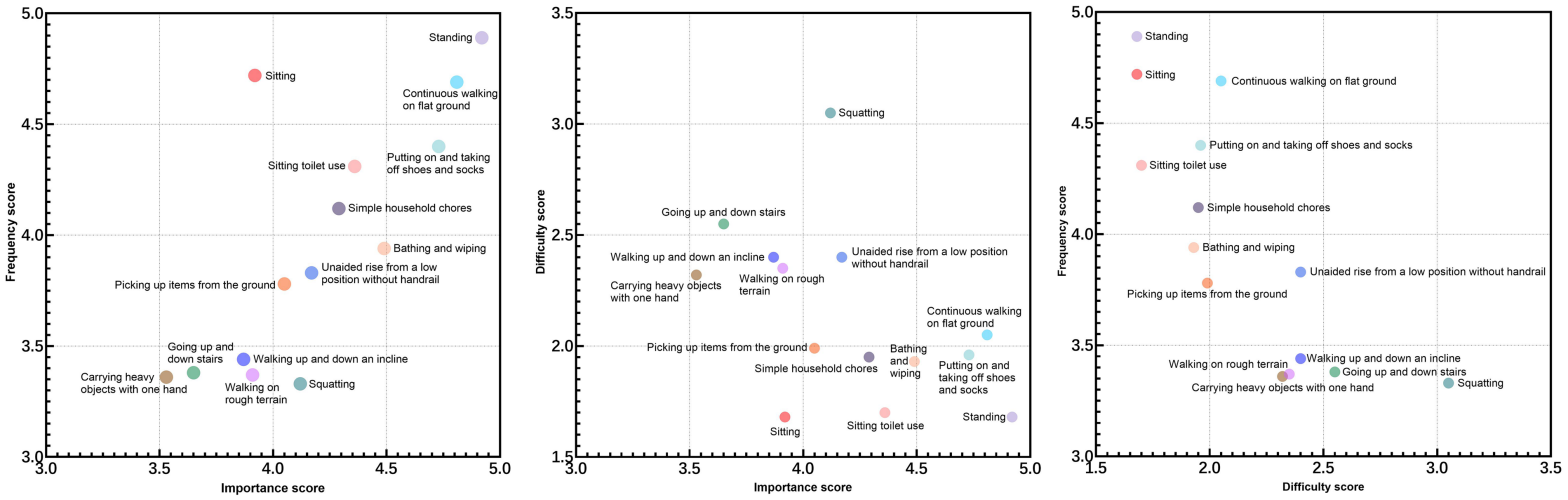
Scatter plots of activity importance, frequency, and difficulty.

Among the 14 activities, a strong positive correlation was observed between importance and frequency, as well as between difficulty and frequency. This indicates that patients tend to persist in performing activities they deem important, despite incomplete knee function recovery. Daily functional demands may drive continued participation despite residual limitations (Figure 2).

Although TKA effectively relieves pain, squatting remained the most challenging activity, with more than three-quarters of patients reporting persistent difficulty. As shown in the scatter plot, the difficulty score for squatting was substantially higher than that of the other 13 activities.

Age-stratified analysis (<65 years, 65–75 years, >75 years) revealed that male patients reported significantly higher activity frequencies in six items: Continuous walking on flat ground, Bathing and wiping, Unaided rise from a low position without handrail, squatting, Picking up items from the ground, and Carrying heavy objects with one hand. In contrast, female patients reported a higher frequency of Sitting toilet use. These findings suggest that gender-specific lifestyle habits may contribute to the uneven distribution of activity frequency (Supplementary Figures 1–3).

**Figure 3:**
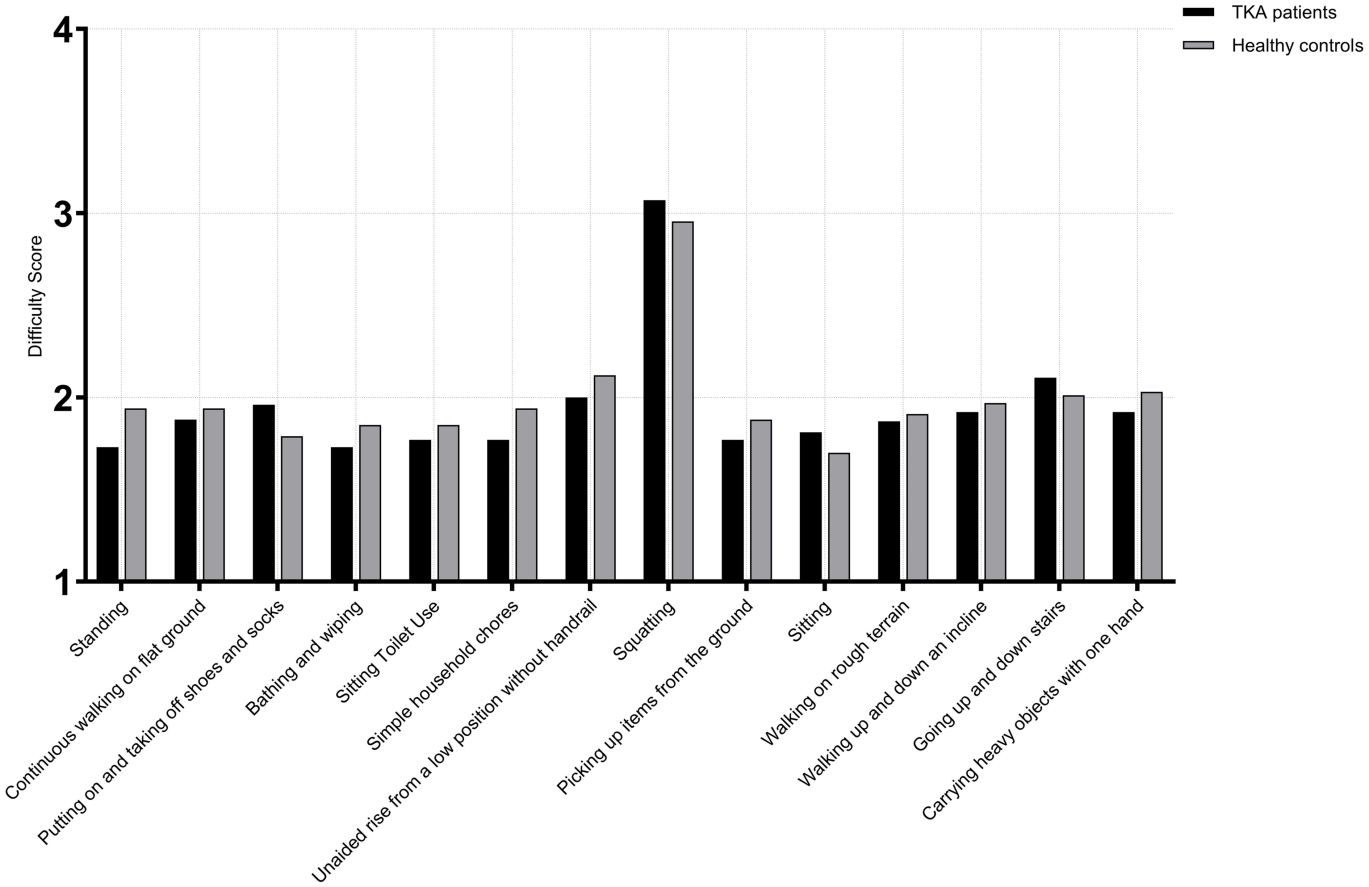
Activity difficulty scores in male TKA patients compared with healthy controls (<65 years).

**Figure 4:**
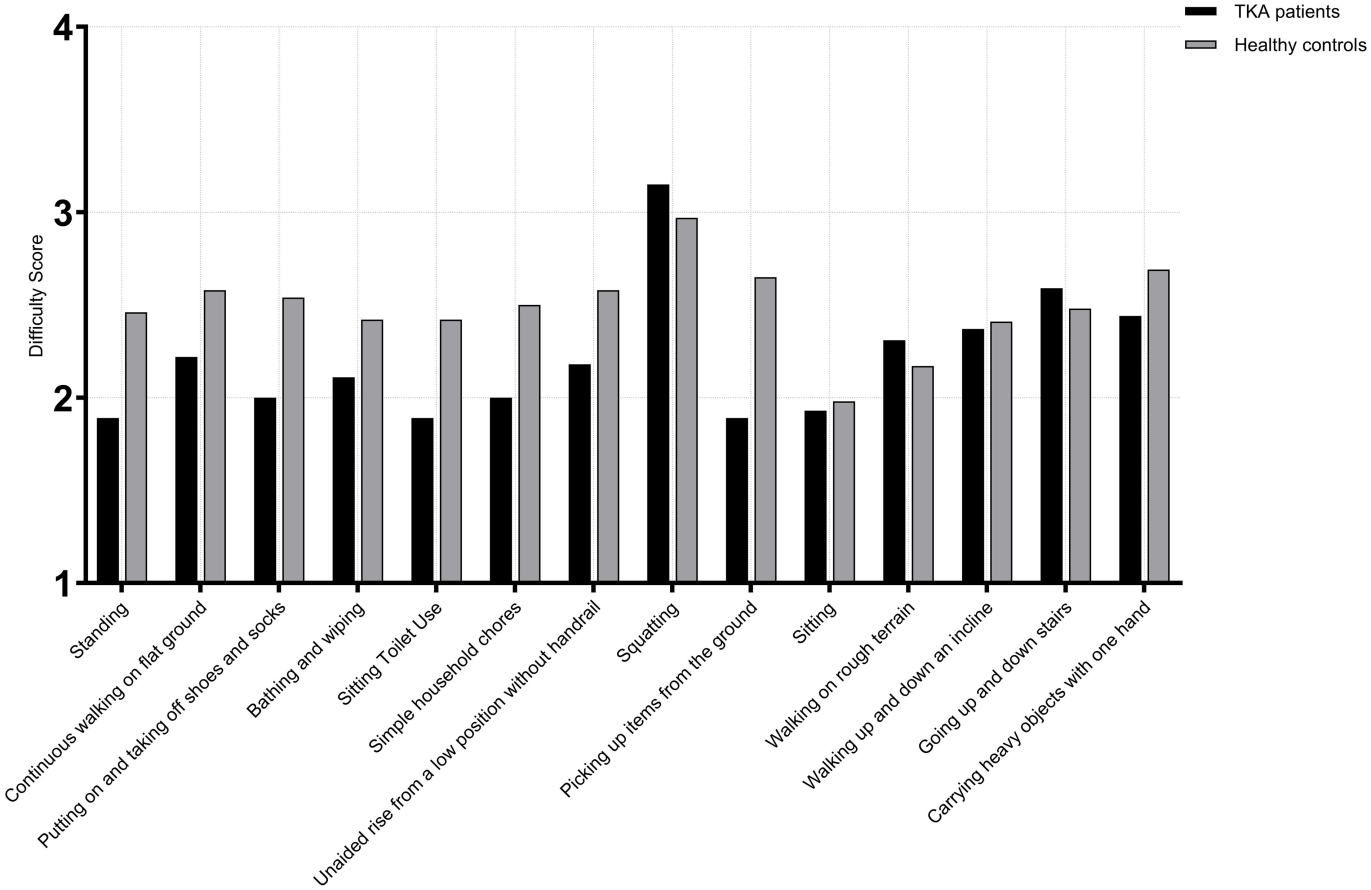
Activity difficulty scores in male TKA patients compared with healthy controls (65-75 years).

**Figure 5:**
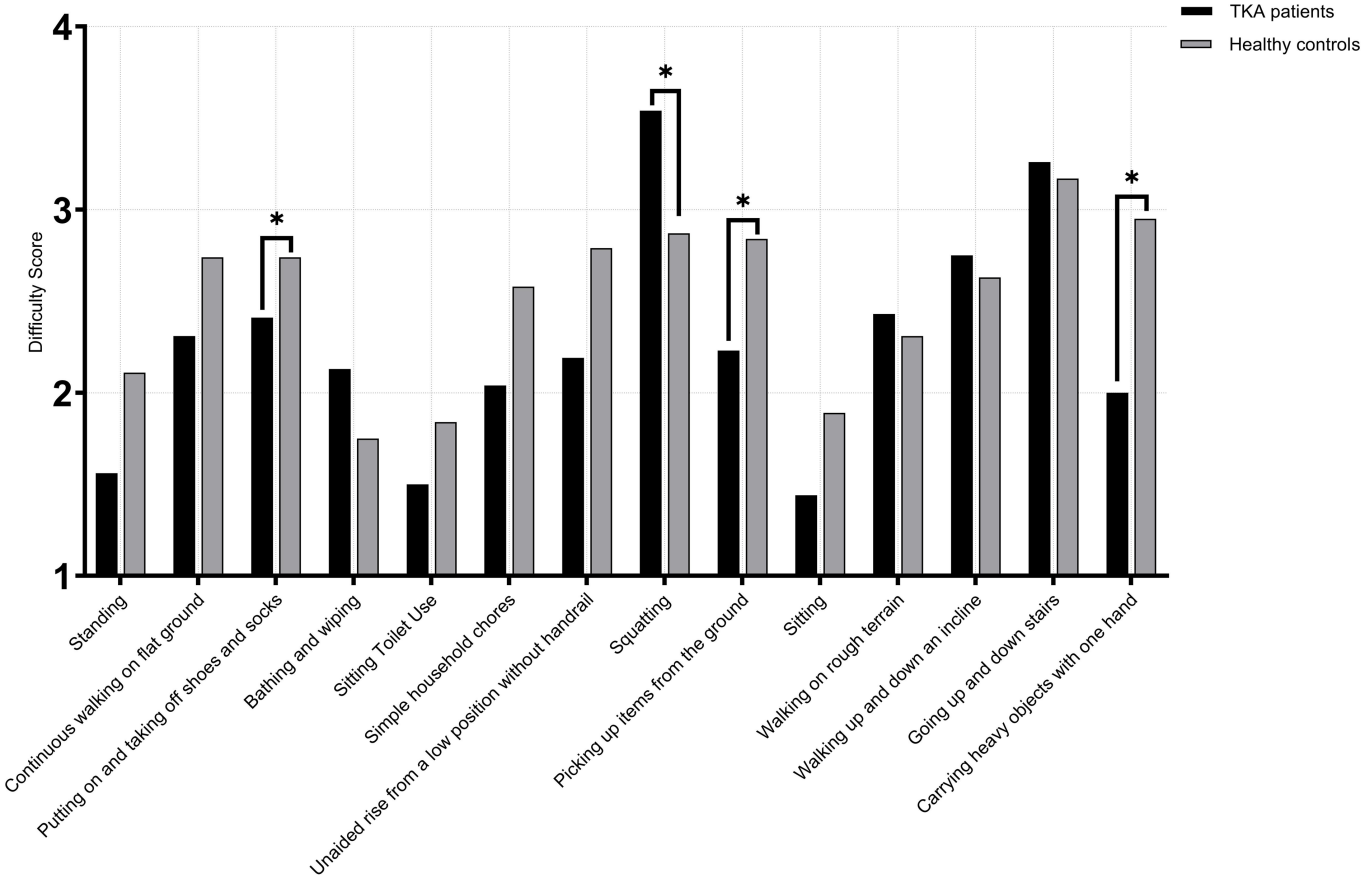
Activity difficulty scores in male TKA patients compared with healthy controls (>75 years).

At 1 year postoperatively, comparison between male TKA patients and age-matched healthy males showed no significant differences across all 14 high-frequency activities in the <65 years (TKA: *n* = 71; control: *n* = 68) and 65–75 years subgroups (TKA: *n* = 118; control: *n* = 114) (*p* > 0.05). However, in the >75 age subgroup (TKA: *n* = 46; control: *n* = 45), For putting on and taking off shoes and socks, TKA patients demonstrated poorer performance than healthy controls (*U* = 730.0, *p* = 0.008, *r* = 0.25), indicating a modest functional reduction. For squatting, TKA patients showed markedly lower capacity (*U* = 1627.0, *p* < 0.001, *r* = 0.49), typically presenting approximately one-point lower scores. For picking up items from the ground, TKA patients again performed significantly worse (*U* = 715.0, *p* = 0.009, *r* = 0.27), consistent with an approximate one-point deficit compared with healthy individuals. The largest difference appeared in carrying heavy objects with one hand, where TKA patients demonstrated substantial impairment (*U* = 46.0, *p* < 0.001, *r* = 0.82), reflecting a clear disadvantage in load-bearing tasks. No other activities showed significant differences between the two groups(*p* > 0.05) (Figures 3–5).

**Figure 6:**
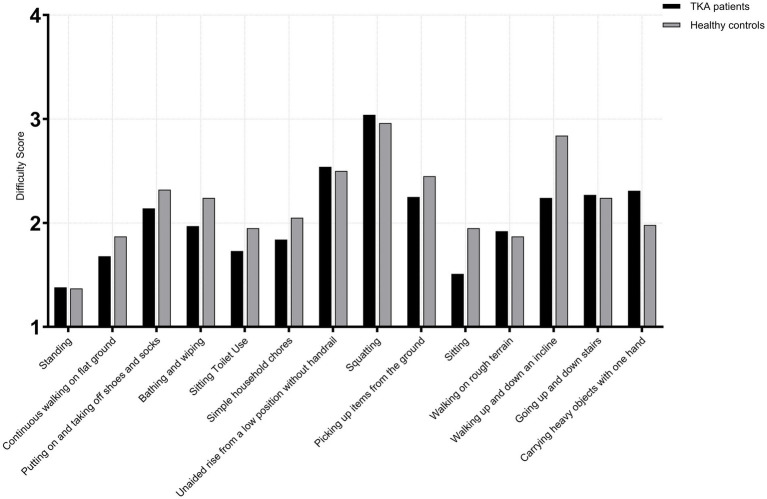
Activity difficulty scores in female TKA patients compared with healthy controls (<65 years).

**Figure 7:**
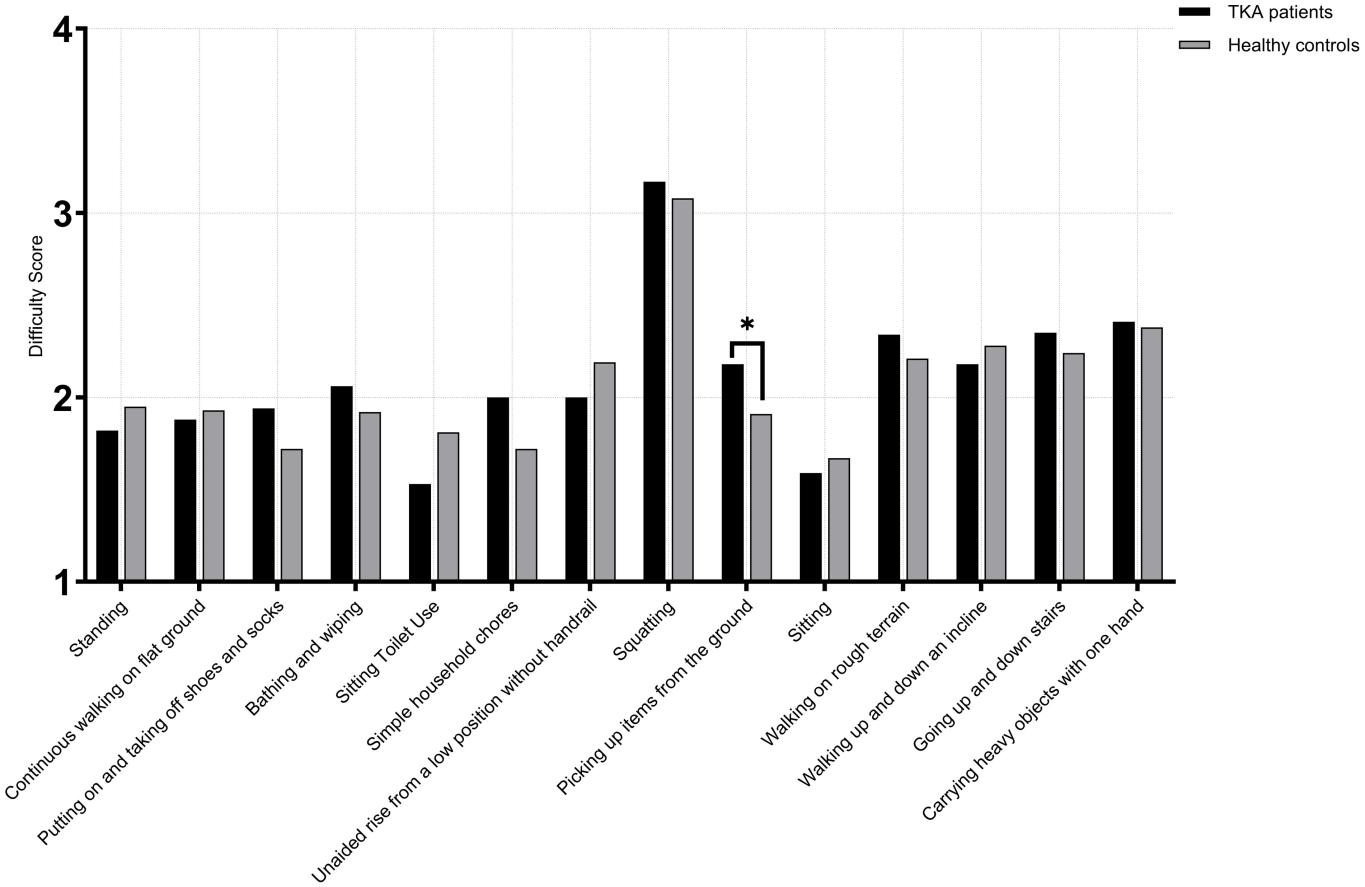
Activity difficulty scores in female TKA patients compared with healthy controls (65-75 years).

**Figure 8:**
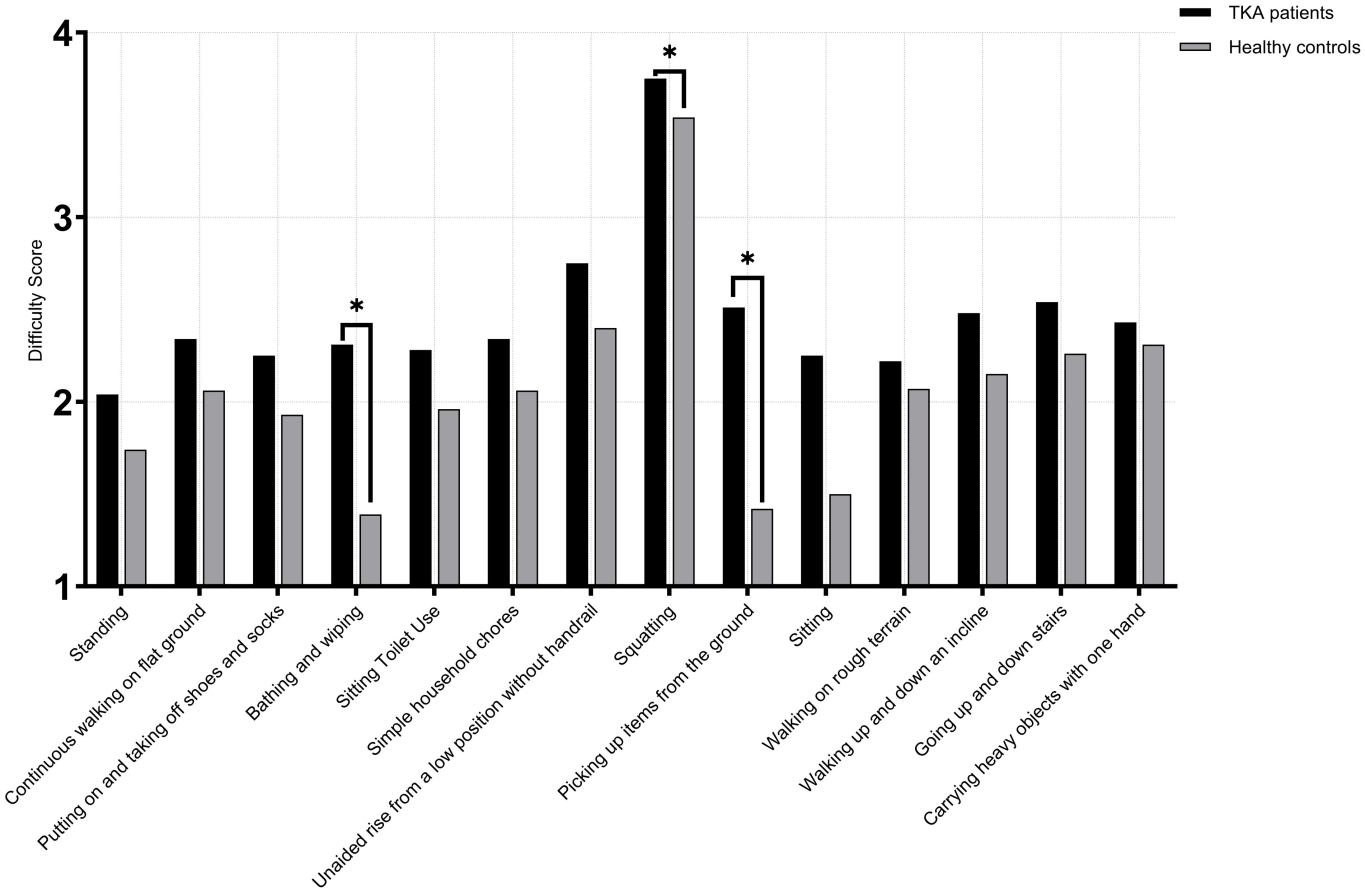
Activity difficulty scores in female TKA patients compared with healthy controls (>75 years).

Among female TKA patients and healthy age-matched female controls, no significant differences were observed across all 14 high-frequency activities in the <65 years subgroups (TKA: *n* = 143; control: *n* = 135) (*p* > 0.05). In the 65–75 years subgroups (TKA: *n* = 239; control: *n* = 224), a significant difference was observed for picking up items from the ground (*U* = 32,386.5, *p* < 0.001, *r* = 0.18). No other activities demonstrated significant between-group differences (*p* > 0.05). In the >75 years subgroup (TKA: *n* = 96; control: *n* = 89), significant differences were observed in bathing and wiping (*U* = 6,930.0, *p* < 0.001, *r* = 0.54) and picking up items from the ground (*U* = 7,166.5, *p* < 0.001, *r* = 0.58), with TKA patients demonstrating markedly poorer performance. A smaller yet significant difference was also noted for squatting (*U* = 4,931.0, *p* = 0.048, *r* = 0.13). All other activities showed no significant differences between groups (*p* > 0.05) (Figures 6–8).

At 1 year post-TKA, patients showed good recovery in low-impact, low-flexion basic activities, with no significant difference in perceived difficulty compared to healthy controls. However, patients over 75 exhibited significant limitations in high-flexion, high-impact, or high-load activities like squatting and picking up items from the floor. These areas represent critical challenges in postoperative recovery and priority targets for rehabilitation (30).

## Discussion

This study identified 14 culturally relevant, high-frequency functional activities that reflect daily knee demands in Chinese TKA patients. Most patients achieved functional performance comparable to age-matched healthy individuals 1 year after surgery, particularly for low-impact daily tasks. However, elderly patients (>75 years) demonstrated persistent limitations in high-flexion activities such as squatting and picking up items from the floor. These findings highlight meaningful recovery in basic mobility but emphasize the need for age-specific rehabilitation targets. The knee function items identified in this study serve as a foundation for the development of culturally adapted knee function assessment instruments tailored to Chinese patients (31–33).

Existing Western PROMs often fail to capture culturally specific functional demands in Chinese patients, such as frequent squatting and floor-level activities. Similar observations have been reported in Asian populations, where deep knee flexion remains highly valued post-TKA. Our results confirm this relevance and support previous calls for region-specific outcome tools that reflect lifestyle and functional priorities in Asian elderly adults (34–37).

Considering that most Chinese TKA patients are elderly and have limited education, the questionnaire was simplified based on patient and expert feedback to ensure clarity and feasibility (38). On average, it took 12 min to complete, indicating high practicality. Guidance by trained research staff minimized misunderstanding and improved data quality. These measures contributed to a user-friendly and culturally sensitive assessment framework suitable for clinical settings.

Demographic factors significantly influenced recovery outcomes. In our cohort, 64.3% were female, consistent with the epidemiology of knee osteoarthritis in China, where TKA is more common in women (39). Gender differences in hormone levels, muscle strength, and health awareness may partly explain functional disparities. The mean BMI of 25.39 kg/m^2^ highlights obesity as a modifiable risk factor, underscoring the need for weight management before and after surgery (40).

Although most patients regained near-normal function 1 year after surgery, those over 75 years experienced persistent difficulty in high-flexion tasks such as squatting or picking up items from the ground (41, 42). These limitations likely stem from age-related musculoskeletal decline rather than surgical insufficiency. While postoperative pain is effectively relieved, irreversible degenerative changes and reduced neuromuscular coordination may prevent full recovery, emphasizing the need for age-adjusted rehabilitation goals (43–45).

These findings emphasize the importance of individualized rehabilitation, particularly flexion-focused strengthening and balance training in older adults. Clinicians should also provide expectation-aligned counseling to elderly patients to avoid unrealistic pursuit of pre-morbid flexion capacity. Integrating culturally specific functional benchmarks may improve patient satisfaction and guide postoperative monitoring (46).

This study has several limitations. First, it was conducted at a single high-volume tertiary orthopedic center, which may limit generalizability, although the diverse patient population enhances representativeness. Second, functional outcomes were self-reported, potentially introducing recall bias despite standardized data collection procedures and trained staff support. Third, as a cross-sectional study, causality between demographic factors and functional outcomes cannot be inferred. In addition, comorbidities and postoperative rehabilitation variation were not fully quantified, which may confound subgroup differences. Future longitudinal studies with detailed comorbidity profiles and standardized rehabilitation monitoring are warranted to minimize residual confounding.

In summary, this study identified culturally relevant, high-frequency daily activities that reflect functional demands among Chinese TKA patients and demonstrated that most individuals regain near-normal knee function 1 year after surgery. However, meaningful limitations persist in adults over 75 years, particularly in deep-flexion and load-bearing tasks, highlighting the importance of age-appropriate rehabilitation goals and expectation counseling.

Future research should focus on longitudinal validation of the identified items, psychometric testing, and integration of this assessment framework into clinical pathways to support individualized rehabilitation and long-term functional monitoring.

## Data Availability

The original contributions presented in the study are included in the article/Supplementary material, further inquiries can be directed to the corresponding authors.

## Supplementary material

SUPPLEMENTARY FIGURE 1 Frequency scores of functional activities in male and female TKA patients (age <65 years).

SUPPLEMENTARY FIGURE 2 Frequency scores of functional activities in male and female TKA patients (age 65–75 years).

SUPPLEMENTARY FIGURE 3 Frequency scores of functional activities in male and female TKA patients (age >75 years).
