# Supplementary figures and images for: Can Chinese patients restore normal knee function 1 year after total knee arthroplasty?
Source: Front Med (Lausanne). 2025 Nov 17;12:1683601. doi: 10.3389/fmed.2025.1683601 (PMC12665790; doi:10.3389/fmed.2025.1683601)

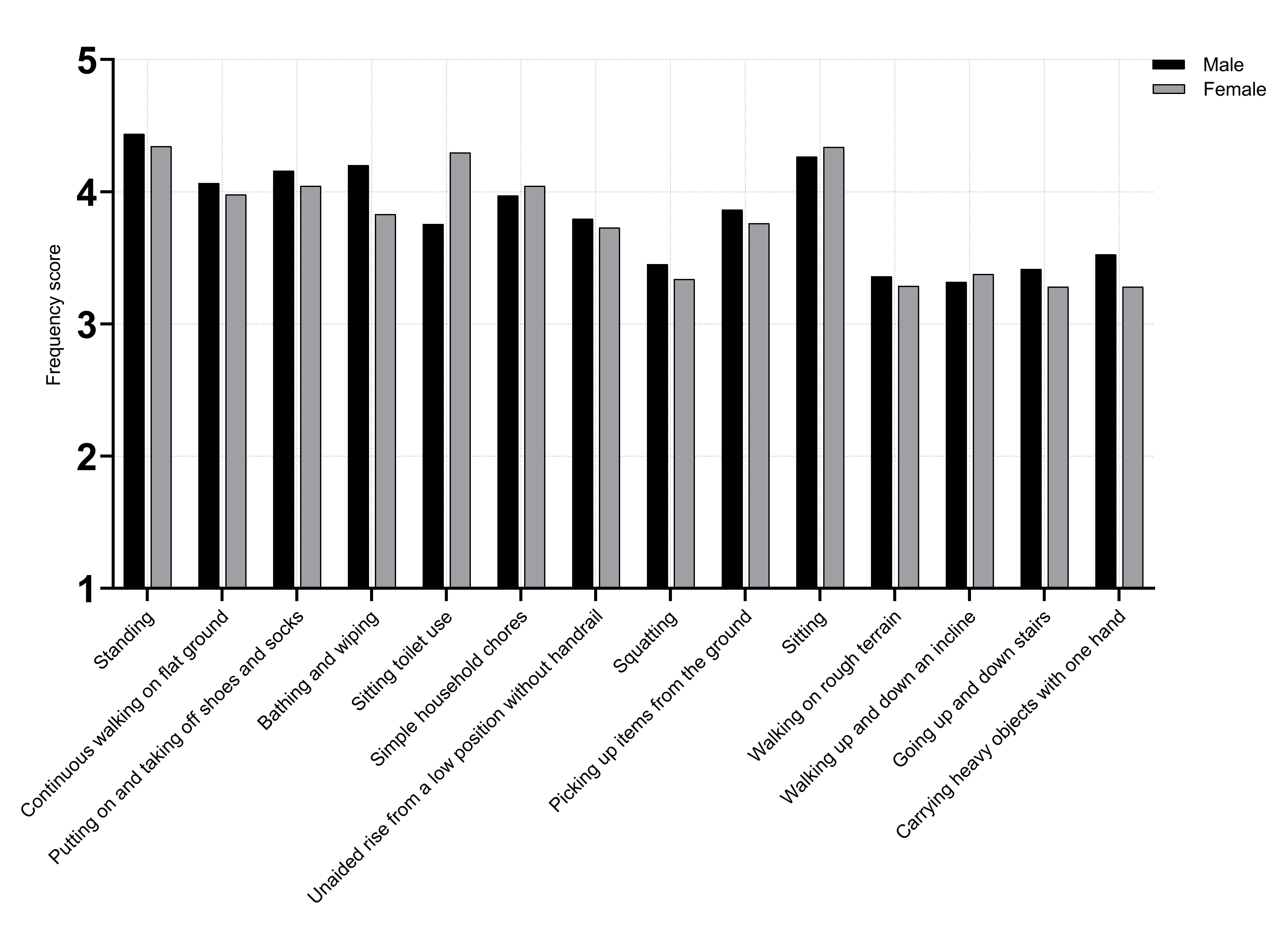

Supplement: SUPPLEMENTARY FIGURE 1 — Frequency scores of functional activities in male and female TKA patients (age <65 years). [file Image_1.JPEG]

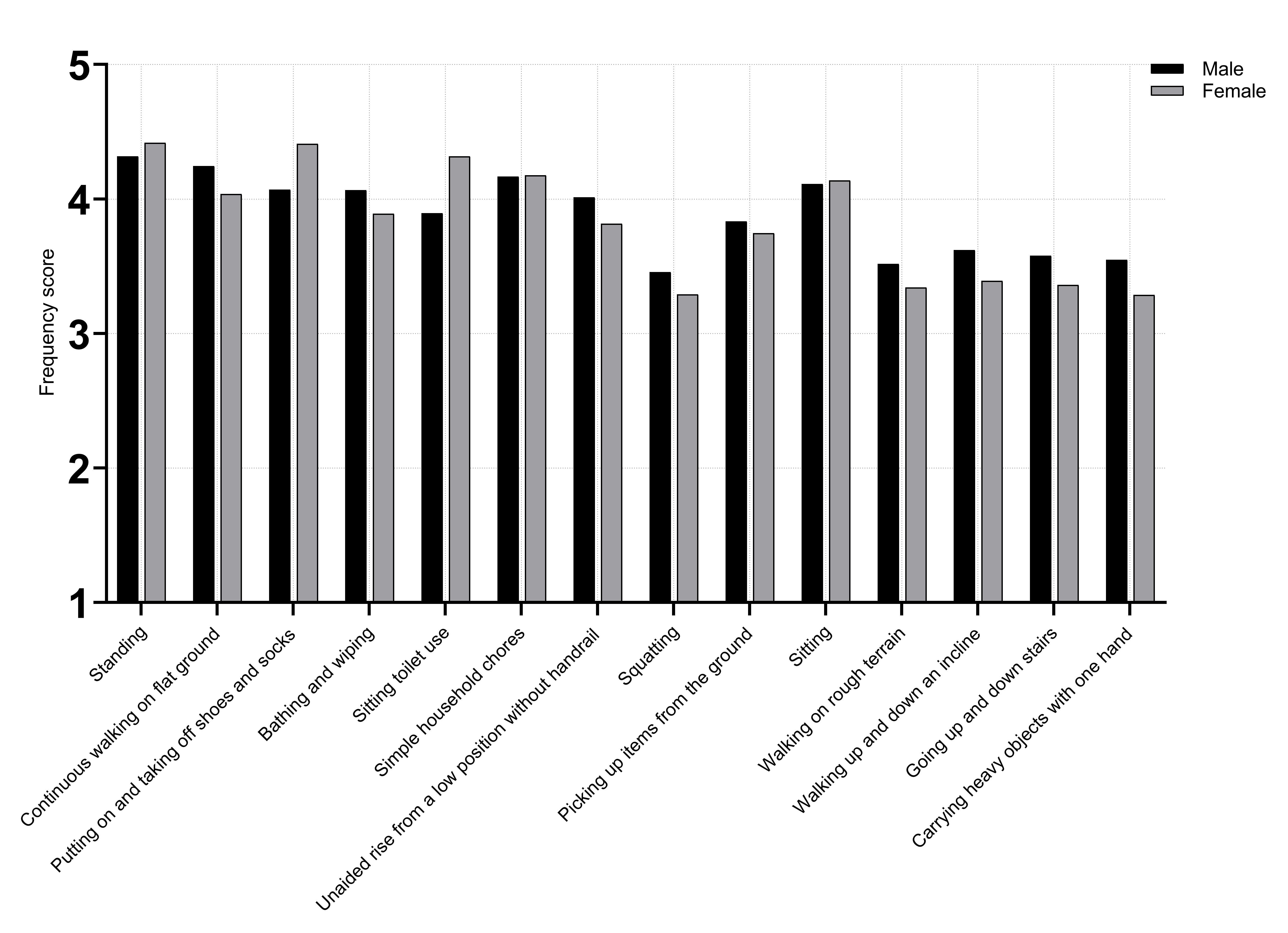

Supplement: SUPPLEMENTARY FIGURE 2 — Frequency scores of functional activities in male and female TKA patients (age 65–75 years). [file Image_2.JPEG]

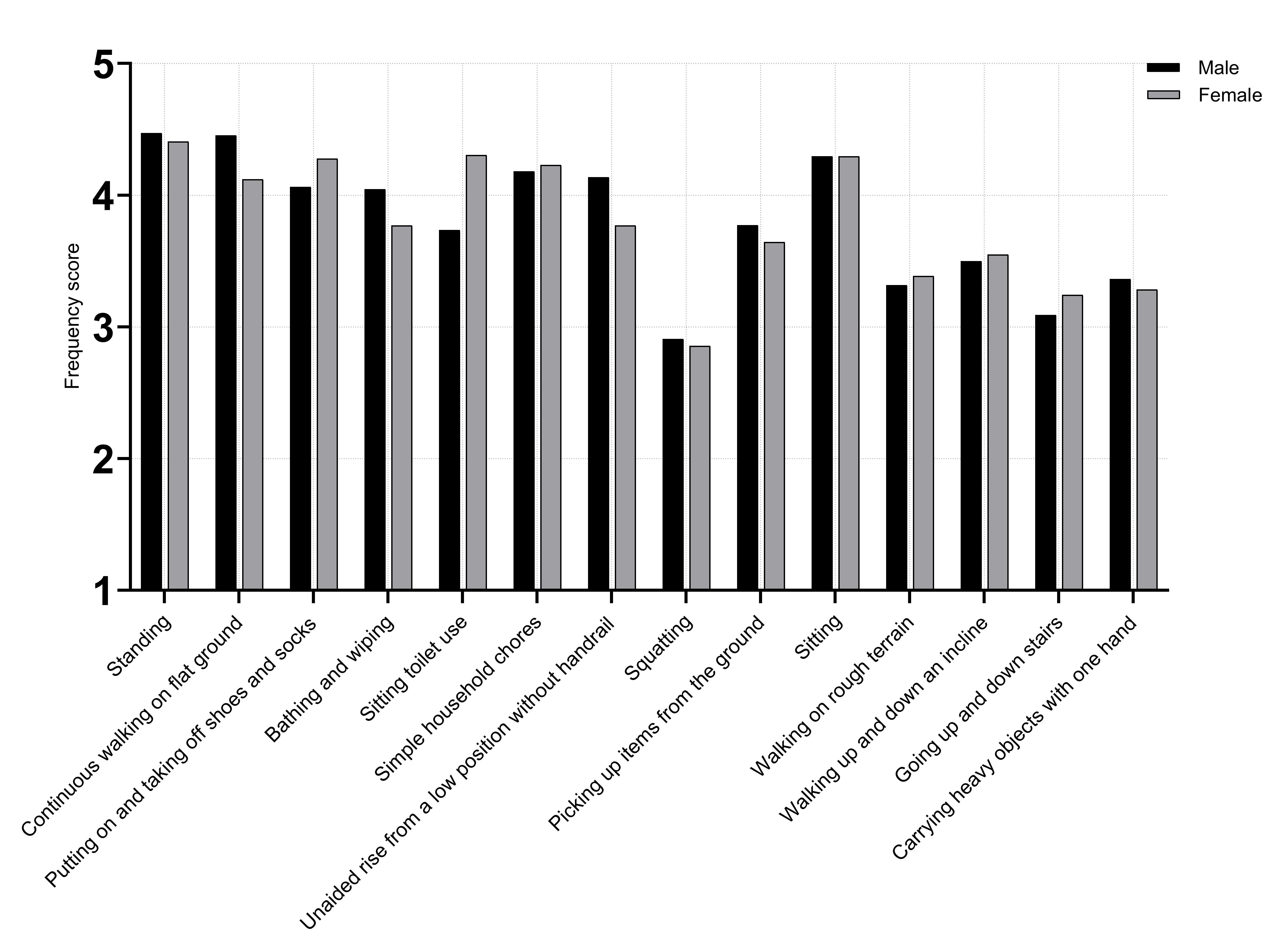

Supplement: SUPPLEMENTARY FIGURE 3 — Frequency scores of functional activities in male and female TKA patients (age >75 years). [file Image_3.JPEG]
